# Hatemongers ride on echo chambers to escalate hate speech diffusion

**DOI:** 10.1093/pnasnexus/pgad041

**Published:** 2023-02-08

**Authors:** Vasu Goel, Dhruv Sahnan, Subhabrata Dutta, Anil Bandhakavi, Tanmoy Chakraborty

**Affiliations:** Department of Computer Science & Engineering, IIIT Delhi, 110020India; Department of Computer Science & Engineering, IIIT Delhi, 110020India; Department of Electrical Engineering, IIT Delhi, 110016India; Jadavpur University, Kolkata 700032, India; Logically, 9-12 Long Lane, London, EC1A 9HA; Department of Electrical Engineering, IIT Delhi, 110016India

**Keywords:** hatemongers, hate speech, echo chamber, information diffusion

## Abstract

Recent years have witnessed a swelling rise of hateful and abusive content over online social networks. While detection and moderation of hate speech have been the early go-to countermeasures, the solution requires a deeper exploration of the dynamics of hate generation and propagation. We analyze more than 32 million posts from over 6.8 million users across three popular online social networks to investigate the interrelations between hateful behavior, information dissemination, and polarized organization mediated by echo chambers. We find that hatemongers play a more crucial role in governing the spread of information compared to singled-out hateful content. This observation holds for both the growth of information cascades as well as the conglomeration of hateful actors. Dissection of the core-wise distribution of these networks points towards the fact that hateful users acquire a more well-connected position in the social network and often flock together to build up information cascades. We observe that this cohesion is far from mere organized behavior; instead, in these networks, hatemongers dominate the echo chambers—groups of users actively align themselves to specific ideological positions. The observed dominance of hateful users to inflate information cascades is primarily via user interactions amplified within these echo chambers. We conclude our study with a cautionary note that popularity-based recommendation of content is susceptible to be exploited by hatemongers given their potential to escalate content popularity via echo-chambered interactions.

**Significance Statement**

This work investigates the spread of online hate speech through the lens of information propagation, user engagement, and polarized consumption/production of information via echo chambers. Our findings establish that hatemongers are way more potent to control the information dissemination over online social networks, even with apparently non-hateful introductions, compared to singled-out hateful posts. This is largely due to such users acquiring a well-connected position on these networks through cohesive engagement with other hateful actors. Unlike merely organized activities, we find that this cohesion among hatemongers is realized via echo chamber formation. We show that the amplified potential of hatemongers to popularize content is controlled via their echo chamber memberships.

## Introduction

The early upheaval of online social networks like Facebook, Twitter, Reddit, etc., to revolutionize the mode of communication and day-to-day information consumption has started to saturate. From the standpoint of end-users as information consumers, their presence in everyday life is now ubiquitous (1). While this has significantly increased worldwide connectivity and information production/consumption, it is *no free lunch*. In the past decade, the world has observed a staggering rise in polarization (2), abusive content, and misinformation dominating the online social space (3–5). A recent survey has reported that around 41% of the US population have been on the receiving end of some hateful behavior at least once in their life (6). Furthering the peril, online hate speech has transcended the virtual to sprinkle vitriol into the real (7–9).

The research community has engaged in this arena with increasing efforts as well. Multiple meta-analyses have suggested a superlinear growth in research related to hate speech in recent years (10–12). Most of these studies seek to *identify* hate speech; some explore the *dynamics* as well (13–16). The latter is particularly of interest for combating the spread of hate speech since only content moderation via flagging, banning, or deleting posts may not be enough in this context (17, 18, 16) (it may often incur threats to the democratic principles (19)). It is unanimously agreed that certain malicious groups take advantage of the apparent anonymity on these platforms to create and propagate hateful content (20–22). However, it is unlikely that a handful of malevolent actors could dictate the large-scale characteristics of such platforms; the inner workings of these platforms (23), reinforced by the real-world social processes (24), should be investigated for how they prepare the breeding ground for online hatemongering. Two separate earlier findings in this context prepare the foundation of our current study. Firstly, the diffusion of information over a platform, whether mediated by hateful actors (users) or via hateful content (posts), exhibits different characteristics compared to their non-hateful counterparts (12, 15). Secondly, in the social science community, it has been conjectured that hateful and extremist behavior might be linked with the formation of *echo chambers* (25, 26)—groups of users who share a strong opinion align themselves in the interaction network in such a way that they are exposed to content correlated to their chosen ideology.

Our study spans over three popular social media platforms: Reddit, Twitter, and Gab—the first one is a strongly moderated discussion forum, whereas the latter two are microblogging sites with partial to no moderation (Twitter is not as carefully moderated as Reddit, whereas Gab is an unmoderated platform to promote “freedom of speech”) (27, 28). Therefore, the chosen social media platforms are expected to cover different aspects of online discussion. We collect and analyze a total of 6.8 million users across these three platforms, covering over 32 million posts and over 0.1 million information cascades.

We start by analyzing information cascades characterized by the hatefulness of the source content as well as of the users introducing them to the platforms. An observation common across all three platforms is that *hate attracts hate*—hateful posts/users cluster around a source post/user more if the latter is hateful. However, there is a remarkable distinction in the importance of the type of content vs. the type of the user in terms of procuring further engagement. We observe that *hateful users are more prepollent than hateful content*. Content posted by a highly hateful user is likely to attract more engagement compared to the same posted by a low-hateful user; even non-hateful posts from high-hateful users tend to catalyze larger cascades, compared to posts from low-hateful users. Upon analyzing the hate characteristics of these cascades, we find that the proportion of hateful participation in the cascade is also larger when the source user is hateful compared to when not. This observation further strengthens the claim that mere content moderation is not enough to combat hate speech.

Further investigations unravel the underlying user interaction dynamics, leading to the observed information dissemination characteristics. We notice increasing user hatefulness as we move towards the network cores. This, along with the observed affinity of hateful users to cluster around hateful users (even when they do not post anything hateful specifically) across all the platforms, drives our focus towards investigating the formation of echo chambers and their relation to hatefulness (29–32). To this end, we propose a novel method of echo chamber discovery in online social networks, developed upon the operational formalism of echo chambers defined in (3, 33, 34). Unlike relying on indirect cues of opinion affinity used by earlier works (e.g. URLs), we directly utilize the content posted by users to define “opinion ecology.” We then define an “echo chamber” as a set of users with highly shared ideology (homophily) and selective exposure to an opinion (32, 34).

Analyses of the echo chambers discovered using this method empirically validate the hitherto conjecture that *hateful behavior over online social networks does intensify through echo chamber formation*. The boost in the volume for cascades originating from highly hateful users is shown to be directly attributed to the user’s affiliation to echo chambers. Furthermore, the cascade participation is strongly biased towards users in echo chambers when the source user is also a member. Finally, we assign a *homogeneity score* to an echo chamber based on the degree of mixing of high-hateful vs. low-hateful users as the constituents—a highly pure echo chamber would primarily consist of either high-hateful or non-hateful users. A strong positive correlation between hatefulness and homogeneity is observed across all three social networks; pure echo chambers are predominantly hateful. However, the homogeneity distribution is skewed for different networks—while Gab exclusively contains pure and predominantly hateful echo chambers, Reddit shows a wider spectrum. We conclude this as further evidence of the interrelation between intense polarization and the spread of hate speech over social networking platforms. Since features like *Top posts*, *Hot topics*, *Trending now*, etc., provided by several platforms rely on ranking posts/topics based on the user engagement they receive and draw the attention of other users to them, echo chamber-driven amplification of hatemonger influence can be a critical factor to keep in mind while designing countermeasures.

## Characterizing hate and echo chambers

### Social networks investigated

We investigate three popular online interaction platforms: Reddit, Twitter, and Gab. The interaction scopes defined for the users on these platforms are very distinct. Reddit is primarily designed as a discussion platform with a very limited scope of sharing already posted content. Also, user interactions on Reddit are governed by numerous user-defined communities *aka subreddits* that predefine the broad topics of discussions with varying degrees of decentralized moderation (i.e. each subreddit has its own set of moderation rules, moderator activity, etc.). Twitter and Gab, on the other hand, are predominantly used as information-sharing platforms via posting and resharing; while there are scopes to reply to a certain post back and forth to construct “discussions,” they are rarer as well as smaller compared to resharing-based cascades. Furthermore, Twitter enforces some degree of content moderation in a centralized manner that has been reformulated and reimplemented multiple times in the past; very often, exclusively hateful tweets from users get deleted early on (35). Gab, on the other hand, is maintained as an alter ego of Twitter with absolute freedom of speech (36).

Reddit is composed of submissions and comments. We connect users based on their commenting behavior on different submissions and comments. We follow the method used in (32) to create our network. A directed link from user *x* to user *y* exists if *y* has replied to a comment or submission from *x*. We collect our data from various controversial subreddits for the year 2019 (e.g. controversy, men’s rights, environment, etc.), covering over 0.8 million posts from over 97 thousand users with more than 0.4 million unique user interactions.

Twitter is made up of posts and retweets. We connect users based on their retweeting behavior on different tweets. We follow the method used by Cinelli et al. (32) to construct our network with tweets within April to June, 2019. A directed link from user *x* to user *y* exists if *y* has retweeted or quote-tweeted a tweet from *x*. Altogether, it covers over 15 million unique user interactions among 67 million users. A total of 315 million tweets are analyzed.

Gab is made up of submissions and comments. Similar to Twitter, users can follow each other. For our analysis, we place a directed link from user *x* to user *y* if *y* has reposted or quoted *x*’s post. The repost and quote work in a similar fashion as retweet and quote tweet, respectively. The collected user network consists of 29 thousand users with over 0.1 million unique interactions through 0.3 million posts appeared within October, 2020 to September, 2021.

Readers may refer to the Materials and Methods and Supplementary Material Appendix, Section 1 for more details about the datasets.

### Identification of hateful content and users

Several studies on large-scale hate speech detection use a predefined set of lexicons to identify a piece of content as hateful (37, 38). However, the applicability of such lexicons can become very limited once the topic of discussion shifts. We refrain from defining hate speech on our own. Instead, we rely on existing hate speech detectors. Identification of hate speech strongly depends on the context under consideration, e.g.type of the event being discussed, time-frame, target of the hate speech being directed towards, discussion forum, etc. To circumvent this, we use an ensemble of multiple classifiers trained using different types of hatefulness datasets. We label the degree of hatefulness of each post based on three different state-of-the-art hate speech detectors—Davidson (37), Waseem (39), and Founta (40). A post is tagged as *non-hateful* when *all* the detectors decide it to be non-hateful. If two or more classifiers find them hateful, then we categorize it to be *highly hateful*, otherwise *medium-hateful*. Further discussion on the detection of hate speech is provided in Supplementary Material Appendix, Section 1.

We further classify each user into one of the three buckets based on the hatefulness as suggested in (41)—**high:** if the user posted five or more hateful posts (medium and/or high), **medium:** if the user posted two or more hateful posts, **low:** if the user posted one or no hateful post.

We illustrate the percentage distribution of hateful posts and users as classified by our method for all three datasets in Supplementary Material Appendix, Fig. S2.

### Cascade characterization

We define the cascade formation based on the predominant mode of user interactions over these platforms. For Reddit, a cascade is an *n*-ary tree, originated by a submission and formed by the recursive commenting against the source submission or other comments. For Gab and Twitter, we consider the retweet/quotation activity to be the progenitor of the cascade trees; the original post/submission is treated as the root, while the subsequent reshares are the descendants. We further delineate three quantifiable, structural properties of a cascade—(i) *volume* of a cascade is the sum of out-degrees for all the nodes in the cascade, (ii) *width* of a cascade is the maximum number of cascade participants all at the same distance from the root, and (iii) *height* of a cascade is the maximum distance from the root to a leaf in the cascade tree.

### Detection of echo chambers

In a nutshell, a set of users within a social network is said to form an echo chamber if they exhibit homophily and selective exposure to opinions/ideologies (32, 34). We build upon these two properties to discover the echo chambers. Most existing methods employ indirect signals, such as referring to some URLs that have been already identified for ideological leaning to define the opinion affinity of users and use that to discover echo chambers (31, 34, 42, 32). Since a substantial number of posts/comments either do not contain URLs (43) or are not annotated for opinions, such methods are limited in exploring dynamically evolving social interactions. Instead, we directly use the textual content in the posts. We make topical clusters of user content using our customized topic modeling technique (which is inspired by Angelov (44)) and extend these clusters to groups of users based on the social interaction network created from the dataset (see Materials and Methods, and Supplementary Material Appendix, Section 2 for more details). Our method is completely automated and needs no external annotation of any sort.

## Results

Our analyses of user influence to spread hate revolve around three primary characterizations of user interactions—How do different users spread information via cascading along the network? How do hateful users organize themselves in the network? How is the formation of echo chambers entwined with hateful behaviors?

**Fig. 1.**
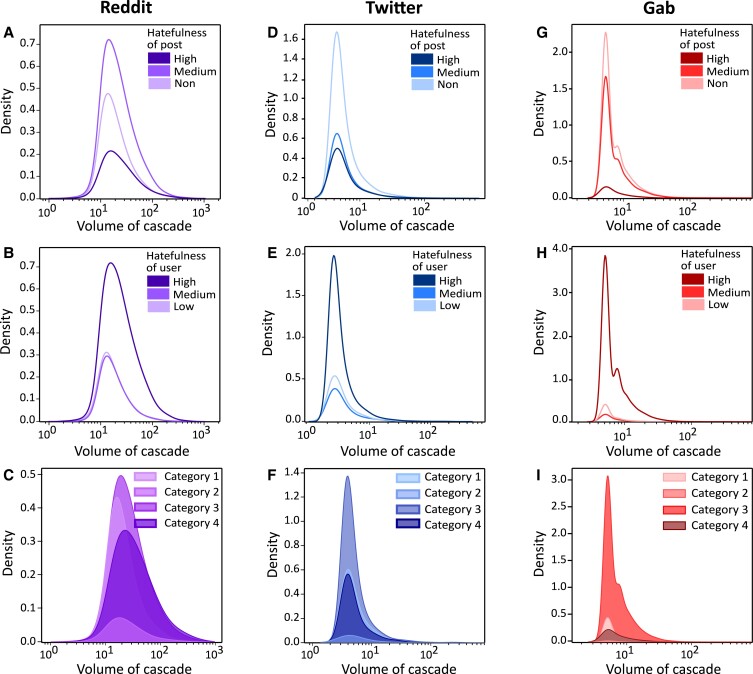
Hateful users are more potent information spreaders. Volumetric density distribution of cascades originating from posts of different hatefulness (subplots A, D, G) vs. posts from users with different hatefulness (subplots B, E, H) are presented; for a given value of cascade volume on the *x*-axis, the corresponding *y*-value denotes the density of cascades corresponding to that volume; alternatively, the area under the curve for certain *x*-interval denotes the fraction of cascades having volume within that interval. For all three networks, posts from highly hateful users are more likely to produce larger cascades. We further analyze the volumetric distribution of cascades originating from hateful users segregated on the basis of hateful posts (subplots C, F, I). Here, **Category 1** represents a non-hate post from a low-hate user, **Category 2** represents a high-hate post from a low-hate user, **Category 3** represents a non-hate post from a high-hate user, and **Category 4** represents a high-hate post from a high-hate user. In all three networks, low-hate content posted by highly hateful users tend to breed largest cascades.

We start with comparing the volumes of the cascades for different degrees of hatefulness (high, medium, and low) of the source posts and the users who posted them (see Supplementary Material Appendix, Fig. S3 for the overall distribution of volume of cascades for the three datasets). Since the cascade volume comes in a distributed array of values, we show the density distribution over different values of volume. Fig. 1B, E, and H suggest that when a highly hateful user writes a post over any of these three networks, the resulting cascade is likely to reach a higher number of users, the Kolomogorov–Smirnov (KS) statistics being 0.198 for Reddit (*p*-value <0.001), 0.147 for Gab (*p*-value <0.001), and 0.049 for Twitter (*p*-value =0.02) (see Supplementary Material Appendix, Section 4 for more details related to statistical significance tests). To paint a picture of the trend, in Reddit, over 70% of the cascades with volumes more than 50 originate from high-hate source users; this number rises to 78% if we set the lower bound on cascade size to be 350. In the case of Gab, such dominance of high-hate users is even more prominent; they are responsible for 88% of the cascades with size >50. Twitter, albeit less prominently compared to Gab or Reddit, exhibits a similar trend with 56% of the cascades having volume >50 coming out of posts from high-hate users. This correlation between hatefulness and cascade size is not directly observed if we move from the hatefulness of the source users to that of source posts (see Fig. 1A, D, and G). We did not find any significant difference in the cascade volume distributions when categorized by the hate-intensity of the source post in the case of Twitter or Gab (KS statistics are found to be insignificant with *p*-value >0.05); for Reddit, we find a weak yet significant difference (KS statistic being 0.11 with *p*-value <0.001).

We observe further nuances when the degree of information dissemination is compared for different types of source content posted by hateful and non-hateful users (Fig. 1C, F, and I). In case of Twitter and Gab, the density distribution characteristics exhibited by hateful users (Fig. 1E and H) are largely composed of cascades originated by non-hateful posts (**Category 3** in Fig. 1F and I). On both these platforms, non-hateful posts from hateful users are more likely to form larger cascades than even hateful posts from the same category of users. Even on Reddit, if the source user is hateful, the volumetric growth of the cascades does not vary much with the degree of hatefulness of the posts that originated them (**Category 3** and **Category 4** in Fig. 1E). Upon additional analysis, we find that this influence of hateful users is not limited to the volume of the cascade but the fraction of hateful interactions as well. In case of Reddit and Twitter, the fraction of hateful interactions is more when the source user is hateful compared to when it is not (see Supplementary Material Appendix, Fig. S1; KS statistic being 0.869 for Reddit, 0.878 for Twitter, and 0.948 for Gab, all with *p*-values <0.001). For Gab, while the absolute number of hateful interactions is higher for hateful users, the fraction gets skewed due to the larger cascades in this case.

A more nuanced introspection of the interplay between the hatefulness of the users, posts, and cascade growths can be done upon topic-wise analysis. In Supplementary Material Appendix (Fig. S8), we show the distribution of hatefulness and cascade volume for different most-occurring topics across the three platforms. It can be readily seen that the point of hatefulness concentration (low, medium, or high) varies across different topics for different platforms, pointing towards the topical dependence of hateful behavior observed in prior studies (15). For topics related to anti-abortion or pro-life, we see that the distribution obtains a peak in Gab but the same is not observed for Reddit. This observation points towards the political inclination of users posting on the respective platforms, and the content they like to engage with.

An opposite question that one may ask at this point is whether or not the role of user hatefulness in information dissemination is actually independent of other user attributes (e.g. age of the user account (45), follower count (43), etc.). Since Reddit does not provide such profile-centric metadata, Gab and Twitter are considered for this sanity check (see Supplementary Material Appendix, Section 4.E for more details on user metadata analysis). Hatefulness of the user shares a very low normalized mutual information (NMI) with the follower count (i.e. the out-degree of the user node in the social network of the platform): 0.034 for Gab and 0.044 for Twitter. Similar patterns are observed in case of user account age as well: 0.045 and 0.055 NMI with user hatefulness in case of Gab and Twitter, respectively. These results signify that the hatefulness of the user is indeed an independent variable and not some artifact manifested by other prominent user attributes in a social network.

We further analyze the structural properties of the cascades in terms of the width and height of the cascade tree (see Supplementary Material Appendix, Figs. S4 and S5). We observe a positive dependence, although in degrees that are platform-dependent, between hatefulness of the source user and the height of the cascade (see Supplementary Material Appendix, Fig. S5) as well as with the width of the cascade (see Supplementary Material Appendix, Fig. S4). Altogether, even though *hate attracts hate* remains true for all three networks, *who is posting* plays an even more dominating role. This is a crucial observation since most prescriptions dealing with hate speech strongly emphasize regulating hateful content; however, a hateful user, if not unchecked, is more likely to disturb the overall harmony compared to isolated instances of hateful content.

**Fig. 2.**
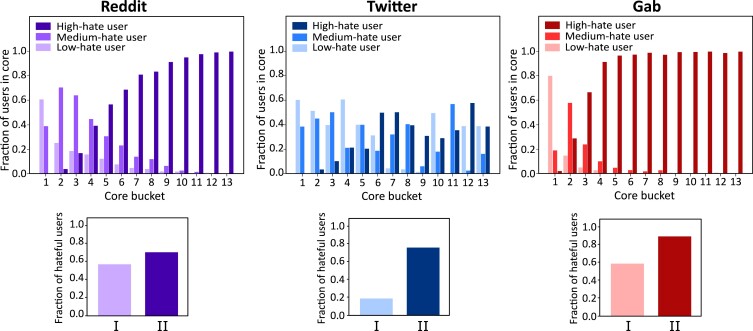
Hateful users seek a greatly connected position in the network with cohesion. (Top) Distribution of hatefulness of users at different order cores (a higher core number indicates greater connectivity of a node). The *i*th value on the *x*-axis represents the *i*th bucket of three cores each (for example, the core bucket 6 represents the user nodes having core values 10–12). For all three networks, users with higher core values are increasingly hateful, signifying a greater connectedness to other nodes in the social networks achieved by the highly hateful users. (Bottom) Fraction of (high- and medium-) hateful users participating in the cascade originated by (I) low-hateful users vs. (II) high-hateful users. A significant affinity is observed among hateful users, pointing towards a possible cohesion among them.

If the actors play a more pivotal role in diffusing information over a network than the content itself, our natural intuition will point towards enquiring about the network organization; highly hateful users should organize themselves in a way that maximizes their influence. This hypothesis is readily justified when we investigate the distribution of hateful users among different depths of the *k*-core decomposition, as shown in Fig. 2(top) (see Supplementary Material Appendix, Section 3 for the details about the *k*-core decomposition). Across all three networks, nodes with higher *k*-core numbers are more likely to be high-hateful (Spearman *ρ* values being 0.68 for Reddit, 0.30 for Twitter, and 0.77 for Gab, all with *p*-value <0.001). Reddit and Gab exhibit a monotonic increase in highly hateful users as the core number increases. For Reddit, highly hateful users tend to dominate the distribution once we surpass 15-cores. For Gab, this transition comes at an even shallower level, probably due to the domination of hatemongers that Gab is infamous for. Twitter, albeit showing a similar overall trend, has some fluctuations in the hatefulness distribution; high- and mid-hateful users start dominating after 18-cores in this case. This organization is reflected in the interaction pattern of the hateful users as well—strong connectivity among the hateful users indicates that they would disseminate information together as well. The cascades initiated by posts from hateful users are more likely to attract other hateful users to participate compared to cascades initiated by non-hateful users (Fig. 2(bottom)), though in this case, Twitter shows the most disparity in user engagement (46), with Reddit being the closest to a balanced scenario among the three. These results also confirm previous studies pointing out the existence of collaborative networks among hate-spreaders (16, 22).

**Fig. 3.**
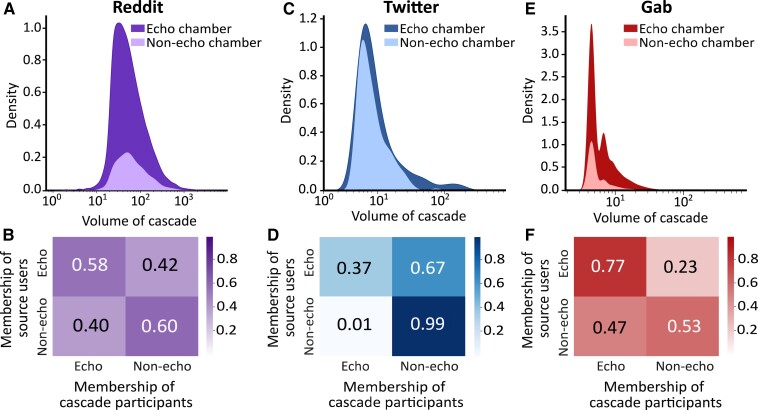
Echo chambers amplify information spreading potential of hatemongers. For each network, A), C), and E) separate out the density distribution of cascade volume for highly hateful source users when they do or do not belong to any echo chamber; particularly for Reddit and Gab, echo-chambered users are responsible for the majority of the high-volume cascades from highly hateful users. B), D), and F) compare the fraction of interactions in the cascades originated by highly hateful echo chamber members coming from other members vs. non-members; here, in Gab and Twitter, echo-chambered users are more likely to participate in cascades originated by fellow echo chamber members.

The previous observation from core decomposition and user engagement characterization points to the fact that hateful users exhibit a unique cohesion. What is left is to verify whether this cohesion can be related to polarization or not. To this end, we employ our proposed method of discovering echo chambers to unfold the further nuances of interactions materialized by the users. We start by dissecting our initial results on cascade growth dynamics (shown earlier in Fig. 1), now aware of the presence of the echo chambers in Fig. 3. Whether or not a highly hateful source user is a member of some echo chambers largely determines the growth disparity occurring among cascades; even highly hateful users who are not members of any echo chambers procure a cascade growth very similar to those originated by low- or medium-hateful users. In all three networks, the density distributions of cascade volumes corresponding to highly hateful users are very close to the fraction of those which have originated by highly hateful users *within* echo chambers. The *p*-value for KS statistic between the cascade volume distributions from all highly hateful source users and those who belong to echo chambers comes to be >0.05 for all three networks, thereby accepting the null hypothesis that these two distributions are indeed the same. We observe significant disparity among the cascade constituent users as well. In the case of Reddit, for example, if the source user is a member of an echo chamber, then 58% of the cascade participants are coming from echo chambers as well; if the source user is not a member of an echo chamber, this number drops to 40% (see Fig. 3B). This disparity is even sharper in the case of Twitter and Gab, as shown in Fig. 3D and F. This is pretty much at par with what we found about the cohesion of hateful users in Fig. 2(bottom)—hate-spreaders are seen to exhibit more affinity to participate in cascades originated by fellow hate-spreaders on both Twitter and Gab compared to that on Reddit.

**Fig. 4.**
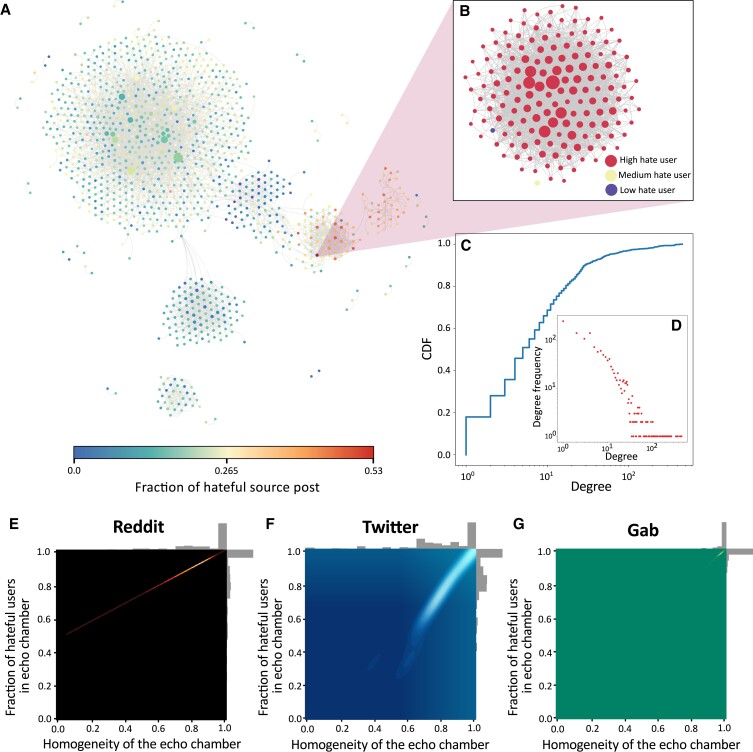
Hatemongers dominate echo chambers. A) A sample distribution of echo chambers in Reddit; each node represents an echo chamber color coded with the fraction of hateful source posts; an edge between two echo chambers denotes shared users (see similar example networks for Gab and Twitter in Supplementary Material Appendix, Figs. S6 and S7). B) A user interaction network within an example echo chamber; each node being a user with edges defined by *reply-to* interactions. C) and D) show the degree distributions of the network shown in A). The distributions of homogeneity vs. fraction of hateful users in echo chambers for Reddit, Twitter, and Gab, respectively are shown in E), F), and G) One would expect a triangular distribution of the echo chambers in the heat maps if there were no relation between the homogeneity and fraction of hateful users present in the echo chambers. Instead, homogenous echo chambers are dominated by hateful users in all three networks.

Up to this point, our findings divulge key roles of hateful users in a social network and how further complexities are integrated into this context with the formation of echo chambers. A sample network of echo chambers (nodes are echo chambers, and edges signify shared users) in Reddit is shown in Fig. 4A; the degree distribution characteristics of the same network are presented in Fig. 4C and D. The inner organization of the users within an example echo chamber is shown in Fig. 4B. Finally, we seek to characterize the echo chambers based on their constituent users. In the context of hate speech, we first put forward a simple definition of *homogeneity* of an echo chamber as:


$$p(E)=\frac{\left|C(E_{\text{H}})-C(E_{\text{N}})\right|}{C(E)}$$

where *E* is an echo chamber described as a set of users; *E*_H_ and *E*_N_ are the set of high/mid-hateful and low-hateful users in *E*, respectively; |⋅| denotes absolute value of scalar; and *C*( · ) denotes the size of a set. In Fig. 4E, F, and G, we plot the variation of the hatefulness of echo chambers with their homogeneity. Generally, we can observe a linear relationship between the two for all three networks. However, the homogeneity distribution of echo chambers is different for different platforms. In Reddit and Twitter, we can find echo chambers residing towards the least homogenous end of the spectrum; the echo chambers in Gab are predominantly skewed towards the most homogenous (and, as a result, most hateful) end. This again is expected given the notoriety of Gab for giving a free pass to hate spreaders (47).

## Conclusion

We presented a comprehensive analysis of hateful behavior on online social networks under the beacon of information dissemination and polarization in terms of echo chamber formation. Upon investigating three popular social networking sites, namely, Reddit, Twitter, and Gab, we observed multiple intriguing patterns of the spread of hate speech. We established that once posted on a network, hateful content tends to provide an assembly point for further hateful behavior; however, the hatefulness of a user plays a more dictating role in this regard compared to a single content that is hateful. Within all three networks, posts from hateful users tend to engage more users over time, both in terms of total volume as well as the degree of hatefulness, even when the source post is apparently non-hateful. This corroborates previous claims that the combat mechanisms of online hate should not be centered around content moderation only.

Our findings suggest that the observed influence of hateful users in terms of cascade growth can be linked to the fact that they are usually placed within the deeper cores of the networks indicating a greater connectedness with other users. We observed cohesion among the hateful users, the degree of which varies for different networks. To check whether this cohesion results from the organized activity, we proposed a fully-automated method to detect echo chambers in networks based on selective exposure to ideology and homophily. Not to our surprise, we found that the cascade growth dynamics linked to highly hateful users consist of source users belonging to some echo chambers. Following the echo chamber normative, these cascades, in turn, largely consist of participant users from echo chambers. Across all three platforms, we found that echo chambers are strongly biased towards hateful members—the homogeneity of echo chambers, defined as the degree of mixing between hateful and non-hateful users, is highly skewed towards the hateful end. To the best of our knowledge, this is the very first empirical validation of previously made conjectures linking hate-mongering to echo chambers (48, 49). These findings might point toward a shortcoming realized by the content recommendation algorithms of most current social networking platforms. Most of the popular online social networking platforms promote content based on their popularity, reinforcing their already acquired popularity. Hatemongers might be able to exploit this by their strategy to popularize content. Since the boost in the influence of such users is primarily driven by echo chambers, a possible countermeasure can be devised based on conditional content promotion by taking the presence of echo chambers into account.

Our findings primarily connect the existing studies on the recently increasing trend of polarized behavior online and the diffusion of hateful content on the Internet. While we correlated the internal organization of hatemongers and observed disparities in information dissemination, this does not necessarily establish a causal relationship. A chronological observation of echo chamber formation and the evolution of hatefulness over the network may provide the community with the answer to the question: Does hate drive the formation of echo chambers or vice versa? Similar studies may provide further insights into other menaces of online social networks, such as toxicity and misinformation.

## Materials and methods

Here we provide further details on the dataset acquisition, the availability of the data, information about the composition, and other methodological details.

### Data acquisition

For Reddit, we used the Pushshift APIs to fetch data across the year 2019 and filtered out selected subreddits. The description of the subreddits are provided in Supplementary Material Appendix, Table S2. For Gab, we wrote our own custom scraper to fetch data from October, 2020 to September, 2021. The systematic scrapping method is described in Supplementary Material Appendix, Section 1.A.2. For Twitter, we used the same mechanism to extract data as mentioned in (31). We used the Twitter dump available at The Twitter Stream Grab. We collected data within the period of April to June, 2019 and then filtered data based on hashtags (more details in Supplementary Material Appendix, Table S4).

### Empirical datasets

We report the statistics and summary of the dataset in Supplementary Material Appendix, Table S1. The three networks do not index posts into topics by default. For Reddit, we filtered the topics of interest initially using subreddits (see Supplementary Material Appendix, Table S2). For Twitter and Gab, we did the same through the use of hashtags and keywords (see Supplementary Material Appendix, Tables S4 and S3, respectively). The focus of topic selection was to ensure opinionated content and interactions; a good proportion of these topics had been used by previous works as well (e.g. gun ownership, abortion, etc.) (31, 32). The topics also provide a good interest as some of them also have alignment with a user’s political opinions as shown previously by Cinelli et al. (32). For Twitter and Gab, we created links between posts by fetching posts and their retweets, whereas, for Reddit, we constructed the network by fetching the submissions and their corresponding comments. Supplementary Material Appendix, Section 1 contains a detailed description of each dataset along with the hate scoring, characterization methods. The section also includes our approach to validate our hate scoring method, for which we employed three annotators to annotate the hatefulness of the content, and then compared their annotations with the output of our method.

### Detection of echo chambers

We propose an automated method for the detection of echo chambers in a social network, wherein we exploit the recent advancements in Natural Language Processing to our advantage—getting rid of any kind of annotations earlier methods required (32, 34). We use a state-of-the-art sentence encoder (50) over each piece of content and utilize its capability to generate semantically accurate embeddings to cluster similar content. Since the dimension of these real-valued embedding vectors is huge, we apply dimensionality reduction methods to obtain embeddings of smaller sizes. We then perform clustering over these reduced embeddings to extract groups of posts that are essentially discussing a similar theme. The groups or clusters of posts can also be understood as topics. As mentioned earlier, echo chambers exhibit two properties, *ideological homophily* and *selective exposure*; we enforce ideological homophily through the inherent nature of the advance sentence encoder to capture the semantics of a sentence; furthermore, we consider two users to be a part of an echo chamber if and only if they have posted/interacted in multiple common topics on their own accord, which satisfies selective exposure. A detailed description of the method for echo chamber discovery can be found in Supplementary Material Appendix, Section 2.

## Supplementary Material

pgad041_Supplementary_Data

## Data Availability

The data for Reddit can be extracted through the publicly accessible Pushshift APIs. We also make the anonymized version of the dataset for all three networks available on the Open Science Framework (10.17605/OSF.IO/6AZCF). Further description of the dataset is provided in the following sections. The source codes for the analyses conducted in the paper are uploaded on the following Github repository: https://github.com/LCS2-IIITD/Hate-mongerer-and-echo-chambers.
