# Supplementary material for: Hatemongers ride on echo chambers to escalate hate speech diffusion
Source: PNAS Nexus. 2023 Feb 8;2(3):pgad041. doi: 10.1093/pnasnexus/pgad041 (PMC10011877; doi:10.1093/pnasnexus/pgad041)
Supplement: pgad041_Supplementary_Data [file pgad041_supplementary_data.pdf]

Supplementary Information for

Hatemongers ride on echo chambers to escalate hate speech diffusion

Vasu Goel, Dhruv Sahnan, Subhabrata Dutta, Anil Bandhakavi, Tanmoy Chakraborty

Corresponding Author name. Tanmoy Chakraborty
E-mail: tanchak@iitd.ac.in

This PDF file includes:

Supplementary text
Figs. S1 to S8
Tables S1 to S4
SI References

Supporting Information Text

Here we provide details of dataset acquisition, hate scoring mechanism, model specifications, k-core decomposition and significance testing. We also present some additional results supplementing those shown in the main text.

1. Dataset

In this section, we list out specifics for data collection and filtration, their composition, and our hate scoring/characterization methodology. Table S1 contains a detailed description of the three datasets and their user-interaction networks.

Platform	Duration	# posts	$C(U)$	$C(I)$	$\langle k \rangle$	$C(CAS)$
Reddit	Jan'19-Dec'19	851002	97349	464087	9.53	22146
Gab	Oct'20-Sept'21	322561	29066	120211	8.52	22978
Twitter	Apr'19-Jun'19	31500556	6709951	15105559	4.50	59638

Table S1. Detailed statistics of the datasets. We list, for each dataset, the duration of its collection and the number of posts in the final dataset; U, I, and $\langle k \rangle$ denote the set of all users, the set of all unique user-user interactions, and the average degree, respectively, in the constructed user-interaction network, and CAS denotes the set of all post cascades we extract from the dataset. $C(.)$ denotes the size of a set.

A. Data Collection and Filtration. Here we explain the data collection process for all three social media networks individually, along with details of their availability. As part of the filtering process, we mapped key political and social events that took place over the duration of the collection of each dataset. For collecting content based on these events, we performed a keyword/hashtag-based extraction for Gab and Twitter, while subreddit titles and descriptions were enough for Reddit. For each platform, we only considered the user-user interactions (submission-comment on **Reddit**, post-reblog on **Gab**, tweet-retweet on **Twitter**) and the textual content of these interactions for our analyses.

A.1. Reddit. The data dump, extracted from the Pushshift API, contains submissions and their comments from a variety of subreddits and spans the year 2019. We further select subreddits that cater to each side of the socio-political spectrum and contain discussions about major real-world events that took place in 2019. The subreddits selected are explained in Table S2.

Subreddits	Description
MensRights, againstmensrights, MensLib	Discussions entailing men's legal rights and societal issues they face in everyday life. **MensRights** majorly comprises legal rights, but general discussions around their relationship with society are also allowed; **againstmensrights** is generally based on uncovering hate and toxicity in r/MensRights but also in the general men's rights movements; **MensLib** is a more general subreddit, created in an effort to allow positive and open-minded discussions on men's issues.
abortion, prolife, prochoice	Discussions comprising issues around abortion and the recent pro-life/pro-choice movement across the world. **abortion** can be explained as more of a support group for people dealing with abortion and comprises general conversations spanning both spectrums of the pro-life/pro-choice movement; **prolife** and **prochoice** subreddits, as their names suggest, contain discussions around the respective sides of the debate.
environment, climatechange, climateskeptics	Discussions around changes in the environment and their corresponding socio-political movements that are taking place across the world. **environment** contains posts around recent news, information and issues related to the changes in the environment; **climatechange** comprises rational discussions and the consequences of climate change in the present day and the coming years; **climateskeptics** is a subreddit majorly focused on uncovering alarmism and conspiracies in recent discussions on environmentalism.
aliens, area51raid, UFOs	Discussions comprised alien life, the famous US Air Force facility and questions about flying object sightings. **aliens** contains conversations majorly on the possibility of extra-terrestrial life; **area51raid** comprises posts about conspiracies about the highly classified US Air Force facility in Nevada and events to "storm" it together; **UFOs** is a subreddit listing public sightings of unidentified flying objects around the world.
conspiracy, TruthLeaks	Discussions around the most famous conspiracies of all time from across the world. **conspiracy** serves as a thinking ground for any general conspiracy theory and people's opinions on them; **TruthLeaks** contains open-source investigations and evidence to discuss and uncover some of the major conspiracies in play today.

Table S2. List of subreddits in the Reddit dataset we use, along with brief descriptions for each of them. Subreddits are grouped in the list on the basis of similarity in the topics of their discussions.

A.2. Gab. The Mastodon (an open-source social networking service) based microblogging platform is known for its user's far-right socio-political ideology. For extracting the Gab dataset, we identified a set of popular users with high posting activity aligned with the real-world events that happened between October 2020 and September 2021. These users were then used as seed nodes for a custom scraper that we designed to recursively collect users that follow them. Collecting for multiple hops of follow relations, we then built a large social network of users based on followership. The scraper then extracted posting history from the user timelines. Finally, we filtered the data using keyword-based analysis that aligns with the socio-political events we

identified as occurring during the time of our collection. Table S3 explains the keywords that we filtered. The issues identified for filtration majorly comprise topics related to the US politics, with a high percentage of them from the perspective of the far-right supporting population across the world.

Keywords	Description
racism, black, white, arrest, murder	These keywords essentially point to discussions on the prevalence of racism across the United States, fueled by the George Floyd incident; they contain both sides of the debate, i.e., people against racism and white supremacy.
abortion ban, parenthood, texas, prochoice	Discussions majorly over the amendments to abortion laws in various states of the US and their corresponding movements; contain clashes between people from both sides of the debate along with news and information about major incidents.
trump, MAGA, election, biden	Content comprising of the build-up and aftermath of the 2020 US Presidential elections, majorly supporting Donald Trump, indicating the one-sided nature of the platform.
gun laws, ban, shootout	Discussions regarding gun access laws in the US, including the recent movement for supporting the ban of these guns across many states, contain discussions over the various school shootings that took place in the USA.
vaccines, anti-vax, vaxxhappened	Contains opinions of people across the world on the use of vaccines fueled by the COVID-19 pandemic; contains a significant amount of discussions from people identifying themselves as "anti-vaxxers".

Table S3. A brief overview of the keywords in the Gab dataset we use, along with a brief description of each group. Keywords in the list are grouped on the basis of the similarity of the topics they represent.

A.3. Twitter. We followed the approach suggested in (1) to extract a data dump from The Twitter Data Stream. This data dump is a 10% snapshot of the international Twitter feed for the months between April 2019 and June 2019, and the content comprises of a variety of topics ranging from social issues to friendly banter. We performed a hashtag-based analysis over this dump and extract only those tweets (and their retweets) that cater to the real-world issues in discussion during the months the dump belongs to. The events extracted include discussions over the US politics, conspiracies, social rights, and others. Table S4 details some hashtags/keywords in our dataset and brief descriptions for each of them.

Hashtags/Keywords	Description
Trump2020, #MAGA, Dems, #LiberalismIsAMentalDisorder	Tweets discussing the build-up to the 2020 US Elections. The content contains controversies, misinformation, and clashes between the two extreme sides of the US political spectrum.
Gaza, #WeLoveIsrael, #WeStandWithIsrael	These comprise the discussions, support and opposing comments around the Gaza-Israel clashes in 2019, along with the political crisis in Israel.
#metoo, Epstein	Discussions revolving around the famous #meToo movement with people coming out against sexual harassment and hate spread around the topic across the world.
Brexit, #EUElections2019, #PeoplesVote	These keywords are part of the tweets about people's opinions on the Brexit referendum, the controversies around it and clashes between people on both sides of the Brexit debate.
#prolife, #prochoice	Opinions, controversies, and clashes between both spectrums of abortion are the major contributors to these hashtags on Twitter. Moreover, the introduction/amendments of abortion laws across various states of the US gave rise to a majority of the content of this topic.
area 51, #StormArea51	Discussions around the famous "StormArea51" American Facebook event that took place on Twitter, along with controversial conspiracies around the presence of aliens in the US Air Force facility in Nevada.

Table S4. A brief overview of the hashtags/keywords in the Twitter dataset we use, along with a brief description of each group. Hashtags/keywords in the list are further grouped on the basis of the similarity of the topics they represent.

B. Hate Scoring and Characterization. For the majority of our analysis, characterizing the content in terms of the hatefulness is of utmost importance. The current section provides details on how we assign hatefulness score to posts and, finally, characterize posts/users into three degrees of hatefulness each.

B.1. Posts. We subject each post in each dataset to three state-of-the-art hate speech classification systems, namely Davidson's (2), Waseem's (3) and Founta's (4) systems. Each system, based on its paradigm, generates a confidence score for each post, which is used to decide whether that post is found to be hateful or not by that system.

Furthermore, we use these systems' classifications to characterize each post into three buckets of hatefulness – **high:** if two or more systems found the post hateful, **medium:** if one and only one system found the post hateful, and **non:** if none of the systems found the post hateful.

B.2. Users. In order to characterize users into three buckets of hatefulness (low, medium, and high), we extract, for each user, their posts and the hatefulness characteristics, as explained in Section B.1. We then classify each hateful user (must have posted at least one hateful content within the duration of the dataset collection span) as follows – **high:** if the user posted five or more hateful posts (medium and/or high), **medium:** if the user posted two or more hateful posts, and **low:** if the user posted only one or no hateful post, as suggested in (5).

C. Hate Scoring Annotation and Validation. To validate our approach for automated hate speech classification, we sampled a subset of 500 posts from each of the social networks. Three annotators were employed; all of them were in an age-group of 25-30, regular users of these platforms, and served the role of annotators for online toxicity detection previously. Each post was given a score of either 0 (non-hateful), 1 (medium hateful) or 2 (highly hateful) by each of these annotators. For each post, we then took the aggregate of the scores received and round off to the nearest integer to obtain the final annotation score. For annotation we set the following guidelines: 1. We classify abusive or derogatory posts targetting a community, gender, race, religion as highly hateful. 2. Posts that do not fall in above criteria, and express a persons opinion, inform about news, and are not offesnive to any person or community on any rights can be classified as non-hateful. 3. Posts that do not fall in above categories. This does not limit to posts which contain abuses or slangs, but not targetting a community or person directly, such posts are classified as medium-hateful. An inter-annotator agreement of 0.78 Cohen's κ was found. Finally, we evaluate our proposed method of hatefulness scoring using the manually annotated data. The F1 scores of our model foor different platforms are as follows: 0.70 for Reddit, 0.65 for Gab, and 0.72 for Twitter. With the *balanced accuracy metric* provided by Scikit-learn* to handle label imbalances we get the following scores: 0.75 for Reddit, 0.70 for Twitter, and 0.72 for Gab.

Multiple previous studies have pointed toward the fact that hate speech classifiers trained using a specific training dataset annotated to identify specific types of hate speech fail to generalize when the data distribution changes due to shifting in target or time (6, 7). The very definition of hate speech is highly sensitive to multiple factors: considered target of hate (racism, sexism, anti-semitism, etc.), time-frame of the data (different events at different times instantiate different types of hateful discourse), type of content (forum post vs. microblogging vs. long articles) and many more. For example, based on a specific event, hatemongers might come up with disrespectful name-calling terms for their targets. These terms fade out and give space to newer terms based on the ever-happenning world of the online platforms. With such a rapid distribution shift, it is hard to achieve near-perfect classification performance using off-the-shelf classifiers.

D. Distribution through Topical Analysis. In Figure S8, we study the density distribution of degree of hatefulness of source user, source post and the volume of the cascades for some of the top-occuring topics across the three social networks – Reddit, Twitter and Gab. We cover topics ranging from politics to conspiracy theories, and black rights to antisemitism. A close look at these plots reveals that across majority of topics in Gab and Reddit, the user hatefulness density distribution peaks around the highly-hateful users. On the other hand, the same is true for medium-hateful users in Twitter. We observe spikes across both Gab and Twitter for high-hateful users for topics pertaining to the US politics; MAGA, Donald Trump for Twitter and Border laws and anti-abortion laws for Gab. For topics related to anti-abortion or pro-life, we notice that the distribution obtains a peak in Gab but the same is not observed for Reddit. This observation points towards the political inclination of users posting on the respective platforms, and the content they like to engage with. The general trend for the high-hateful users driving the spread of information still persists when observing the distribution of cascade volumes for social networks. However, the magnitude of cascade volumes is intrinsic to the network under consideration.

2. Echo Chamber Detection

We propose a novel method for the detection of echo chambers in a social network. Given that we have access to the interaction network and the content shared between the users, the main idea around which we build our method is to automatically detect interactions based on similar topics and further extract groups of highly-clustered users in the network that take part in said interactions.

Let U be the set of all users in a network, C be the set of all content written on that network, and $C^u \subseteq C$ be the set of all content (posts or comments) written by user $u \in U$ on that network. Finally, let EC be the set of all encoded real-valued vectors, and T be the set of topics as generated using EC, where $\forall t \in T, t \subseteq EC$.

A. Topic Detection. We pass each $c \in C$ through a pretrained natural language encoder to convert each piece of content to a uniquely-encoded real-valued vector $e \in EC_{NLE}$ of size 512 (8). Further, we apply principal component analysis (PCA) and uniform manifold approximation and projection (UMAP) over each vector, to reduce it to a smaller vector $e \in EC$ of size 64.

Let $NLE(\cdot)$ denote the natural language encoder that we use (Universal Sentence Encoder (9), in our case), $PCA(\cdot)$ and $UMAP(\cdot)$ denote the functions for reducing the encoded vectors to a smaller size (10).

$$EC_{NLE} = \bigcup_{c \in C} NLE(c) \quad [1]$$

$$EC = \bigcup_{e \in EC_{NLE}} PCA(UMAP(e)) \quad [2]$$

We represent all of the textual content of the network in the form of these reduced vectors. We then perform clustering (HDBSCAN (11), in our case) on the set EC to find groups of similar content in terms of context and containing similar terms/phrases as detected by the natural language encoder.

* https://scikit-learn.org/stable/modules/generated/sklearn.metrics.balanced_accuracy_score.html

Let $CLS(\cdot)$ denote the clustering algorithm we use, which produces groups of the encoded vectors.

$$T = CLS(EC) \quad [3]$$

Each group represents a collection of posts corresponding to a topic discussed by the users, which we use to further extend to clusters of users discussing common topics. To validate the quality of topical clusters created with the method above, we randomly sample some topics across the three social networks and analyse the content that is classified under them. We observe that the content clustered under a topic is similar in nature. Moreover, not only does the content refer to a similar event in time but also share the same ideology. We have uploaded multiple examples from each sample topic at https://github.com/LCS2-IIITD/Hate-mongerer-and-echo-chambers/tree/main/Sample of Topic Clusters, within the codebase. The files are named with topics identified by the authors, using manual inference of content.

B. Extending Topics to Clusters of Users. In the existing literature, an echo chamber is defined as a group of users who share the same opinion and reinforce their own beliefs (12). We cluster groups of users who share content on multiple topics multiple times. We argue that with this approach, we can segment users in both the criteria of echo chamber detection, i.e., ideological homophily and selective exposure. Since users share content with their own intent, they are being selective to the kind of content they want to react to. And since the topics were clustered using semantic information, the posts in a topic share the same ideology. The users' groups that are initially constructed in this manner are identified as candidate echo chambers. One issue we face is that many users are common across multiple topics, and some of the clusters identified have over 90% similarities in terms of mutual users. We come up with a simple heuristic to reduce the number of unique candidate echo chambers and combine multiple echo chambers if they share a commonality in terms of users or topics above a specified threshold. For our experiments we combine two candidate echo chambers if the Jaccard Coefficient for the set of users belonging to the candidate echo chambers is greater than a threshold (0.7 in our case). The remaining clusters obtained after the reduction are finally classified as echo chambers.

C. Echo Chamber Network. We capture the relations between echo chambers in the form of an echo chamber network. We model this network in the form of an undirected weighted graph $G = (V, E, W)$, where V is the set of echo chambers, E is the set of edges, where $e_{ij} \in E$ denotes the presence of common users between V_i and V_j. W_{ij} denotes the weight of an edge e_{ij}, which is the number of common users between V_i and V_j. Here, we only connect disjoint rumors, i.e., if $\exists e_{ij}$, then $T(V_i) \cap T(V_j) = \emptyset$, where $T(X)$ denotes topics composing echo chamber X. We notice that the networks created are very dense (for Gab and Reddit). To obtain a better visualization, we use a backbone extraction method (13) to get the important links (see Figures S6 and S7).

3. K-core Decomposition

A subgraph is said to be k-core or a core of order k if and only if all the vertices of the subgraph have a minimum degree of k, and it is the largest possible subgraph satisfying that condition.

Mathematically, we can define it as follows. Consider a graph $G = (V, E)$, where V is the set of nodes, and E is the set of edges connecting these nodes. Consider a subgraph $H = (A, E|A)$, where $A \subseteq V$. H is a k-core of G iff $\forall v \in A$: $degree_H(v) \geq k$, and H is the maximum subgraph satisfying the condition.

K-core decomposition is a method in which we partition the graph into multiple cores by varying k. The corresponding cores are nested, i.e., $\forall i < j \implies H_i \subseteq H_j$. It is not necessary for the subgraph to be connected in a core. The method helps us in extracting more central nodes. The higher the k-core number of a node, the more densely it is connected in the network.

4. Significance Testing

A. Volume of Cascades. We hypothesize that the volume of cascades is impacted by the degree of hatefulness of a user and not impacted by the degree of hatefulness of the post. We use the Kolmogorov-Smirnov test to check whether our hypothesis is statistically significant.

To validate the impact of the degree of hatefulness of source users on cascade volume, we consider the continuous distribution of cascades from hateful source users ($F(x)$) and continuous distribution of cascades from non-hateful source users ($G(x)$). We define the null hypothesis as if the two distributions are identical, i.e., $F(x) = G(x)$, and the alternate hypothesis as $F(x) \neq G(x)$.

Similarly, to validate the impact of the degree of hatefulness of source posts on cascade volume, we consider continuous distribution of cascades from hateful source posts ($F(x)$) and continuous distribution of cascades from non-hateful source posts ($G(x)$). The null and alternate hypotheses are defined in the same manner to validate the impact of the degree of hatefulness of a source user.

From our analyses, we conclude that the impact of the degree of hatefulness of a source user on volume of the cascades is statistically significant for all three social networks, with each reporting p-value < 0.02. Hence, we can clearly reject the null hypothesis in this scenario. In contrast, for the degree of hatefulness of source posts, we get p-values > 0.05 for Gab and Twitter, making us unable to reject the null hypothesis.

We conduct similar experiments for cascade width and height for all social networks. We find that impact of the degree of the hatefulness of a source user on cascade width is statistically significant for all three social networks with p-values < 0.05.

For the impact of the degree of the hatefulness of a source post on cascade width, we get p-values > 0.05 for Gab and Twitter, which is synonymous with the scenario for cascade volume. Next, we analyze the impact of the degree of the hatefulness of a source user on cascade height; we get p-values < 0.01 for both Reddit and Gab, which rejects the null hypothesis. Regarding the hatefulness of a source post on cascade height, we get p-value > 0.05 for Gab, indicating that the null hypothesis is accepted.

B. Volume of Cascades of Echo Chamber Users. We hypothesize that the cascade volume distributions from all highly-hateful source users and those source users who belong to echo chambers are different. We use the Kolmogorov-Smirnov test to check whether our hypothesis is statistically significant.

To validate the impact when the source user of a hateful post belongs to an echo chamber, we consider the continuous distribution of cascades from all hateful source users ($F(x)$) and the continuous distribution of cascades from hateful source users that belong to an echo chamber as ($G(x)$). We define the null hypothesis that the distribution of cascades of source users belonging to echo chambers is similar to the distirbution of cascades of hateful users, i.e., $F(x) = G(x)$, and the alternate hypothesis as $F(x) \neq G(x)$.

So, in the case, as our null hypothesis isn't rejected, we can say that the distribution of volumes of cascades with highly-hateful source users that belong echo chambers is similar to the distribution of volumes of cascades from all highly-hateful source users, which is the case that we observe, as we get p-value > 0.05.

In contrast, when we conduct the same experiment by replacing source users belonging to echo chambers with source users not belonging to echo chambers, we observe that we get p-value < 0.05, which rejects the null hypothesis, and we accept the alternate hypothesis.

Hence, we can conclude that when highly-hateful source users belonging to echo chambers post content on social networks, the cascade formed are more similar to the cascades formed by posts from highly-hateful users.

C. Impact of Degree of Hatefulness of a Source User on Fraction of Hateful Interactions when the Source Post is Hateful. We hypothesize that the degree of hatefulness of a user also impacts the fraction of hateful interactions that occur on a hateful post, as shown in Figure S1. To measure if the effect is significant, we run a Kolmogorov-Smirnov test to validate our hypothesis.

To validate the impact of the degree of the hatefulness of a user on the fraction of hateful interactions, we calculate the size of each cascade, the degree of the hatefulness of the source user, the degree of the hatefulness of the source post, and the fraction of hateful interactions in the cascade. We consider the distribution of the fraction of hateful interactions for only a high-hateful post from a high-hateful source user as $F(x)$, and the distribution of the fraction of hateful interactions for a high-hateful post from a low-hateful source user as $G(x)$. We define the null hypothesis as the degree of the hatefulness of a source user having no impact on the fraction of hateful interactions, i.e. $F(x) = G(x)$, and consequently, the alternate hypothesis being $F(x) \neq G(x)$. However, we observe that we get a KS statistic of 0.869 for Reddit, 0.878 for Twitter, and 0.948 for Gab, all with p-values < 0.001. Hence, we can reject the null hypothesis.

D. Core-wise distribution of user hate intensity. To validate the correlation between core number and hatefulness of a user, we calculate the Spearman correlation coefficient between the distribution of core numbers and degree of hatefulness of a user. We get Spearman rho values as follows: 0.68 in Reddit, 0.30 in Twitter, and 0.77 in Gab, all with p-values < 0.001. The values for Spearman p, vary between -1 and 1, with 0 indicating no correlation. Despite the correlation being low for Twitter, we still get high correlation for both Reddit and Gab. We can say that both Reddit and Gab show a monotonic increase in highly-hateful users as the core number increases.

E. User Metadata Analysis. Several studies have established relationships between cascade growth and different attributes of the root user. To establish the validity of user hatefulness as a viable feature of cascade growth, we seek to measure how much information it shares with other cascade predictors. Follower count and age of the user account are two prominent ones among such attributes (14, 15). We compute Normalized Mutual Information (NMI) between a pair of variables; a near zero NMI would suggest independent distributions. Hatefulness of the user shares a very low NMI with the follower count: 0.034 for Gab and 0.044 for Twitter. Similar patterns are observed in case of user account age as well: 0.045 and 0.055 NMI with user hatefulness in case of Gab and Twitter, respectively. We do not elect the following count of the accounts, as accounts of popular users like celebrities, sports personalities and politicians tend to have lower following counts which is also the scenario with people less active on social media.

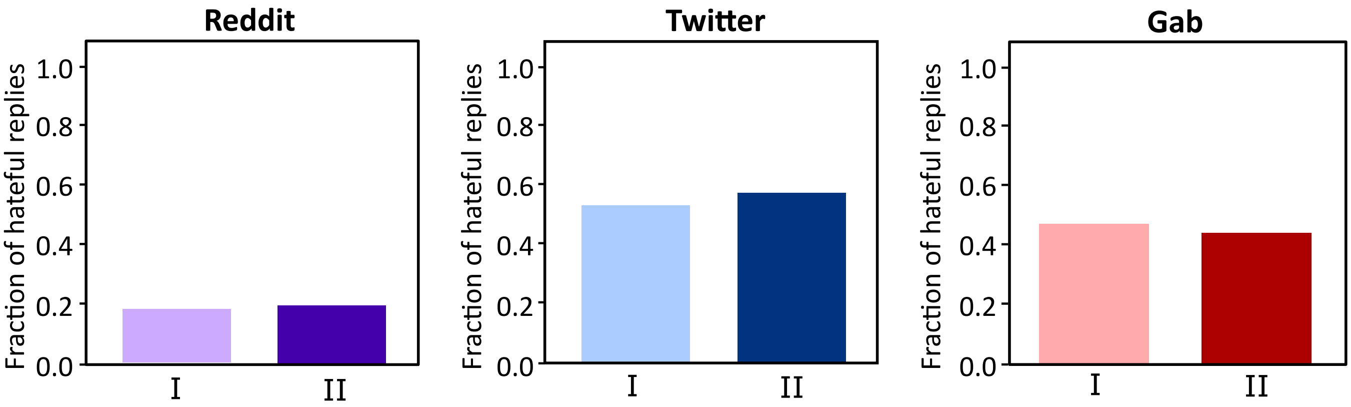

Fig. S1. Fraction of hateful interactions within cascades generated by hateful posts of (I) a low-hate user vs. (II) a high-hate user.

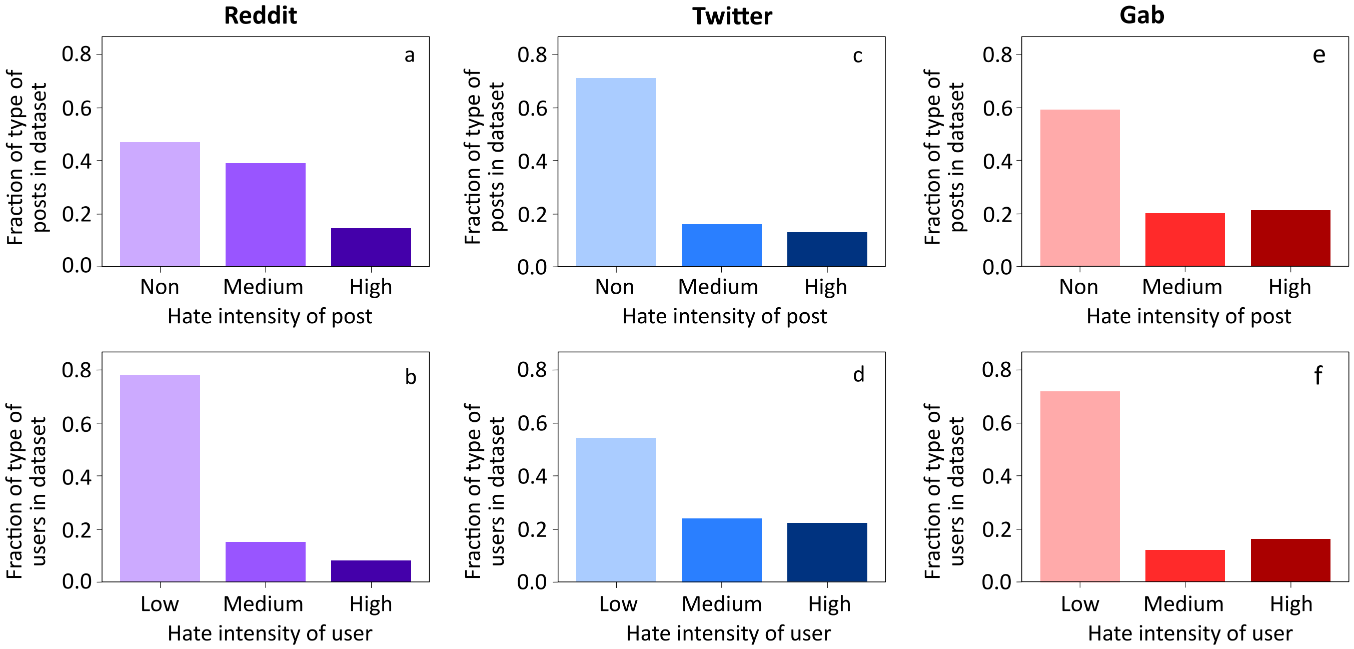

Fig. S2. Fraction of type of posts (subplots a., c., e.) and users (subplots b., d., f.) characterized based on their hatefulness (low, medium, and high).

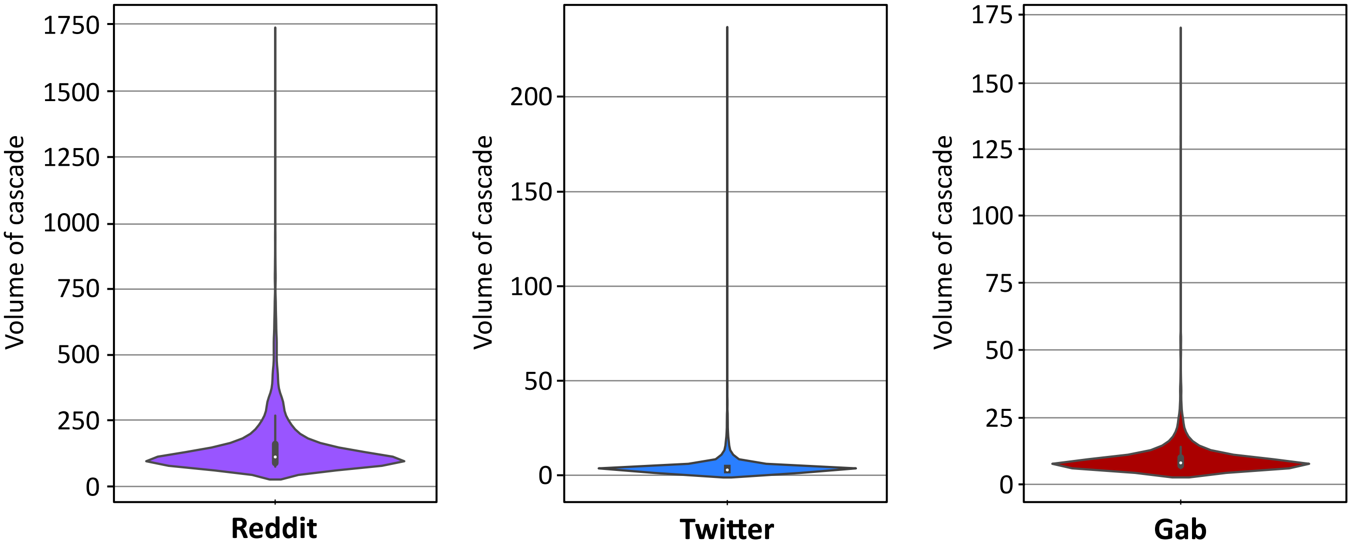

Fig. S3. Distribution of the volume for the top 10% volumetrically largest cascades across all three social networks.

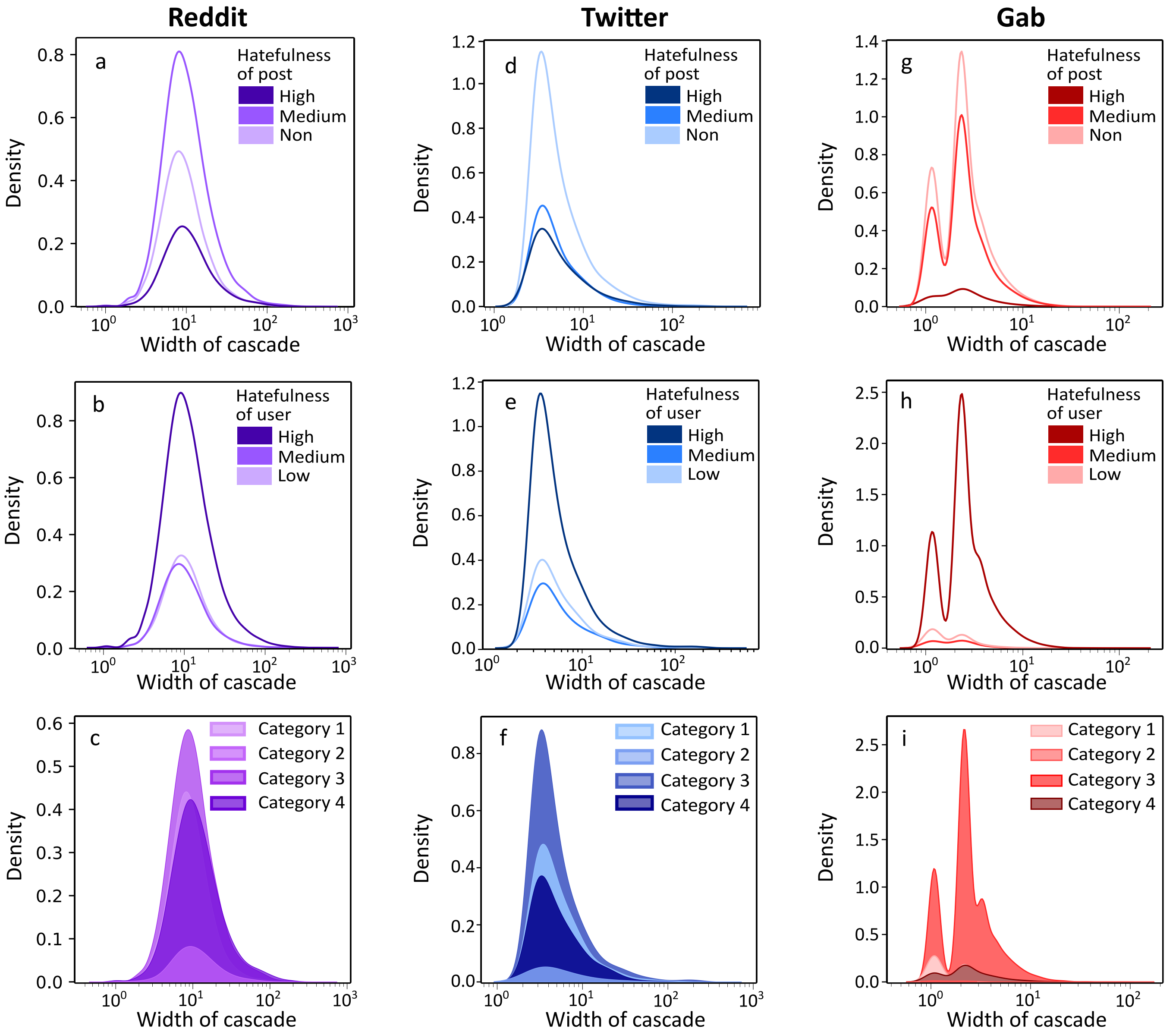

Fig. S4. Width distribution of cascades originating from hateful posts (subplots **a.**, **d.**, **g.**) vs. posts from hateful users (subplots **b.**, **e.**, **h.**); for a given value of cascade width in the x-axis, the corresponding y-value denotes the density of cascades corresponding to that width. For all three networks, posts from highly-hateful users are more likely to produce cascades of larger width. We further present the width distribution of cascades originating from hateful users segregated on the basis of hateful posts (subplots **c.**, **f.**, **i.**). Here, **Category 1** represents a low-hate post from a low-hate user, **Category 2** represents a high-hate post from a low hate user, **Category 3** represents a low-hate post from a high-hate user, and **Category 4** represents a high-hate post from a high-hate user. In all three networks, low-hate content posted by highly-hateful users tend to breed cascades with largest width.

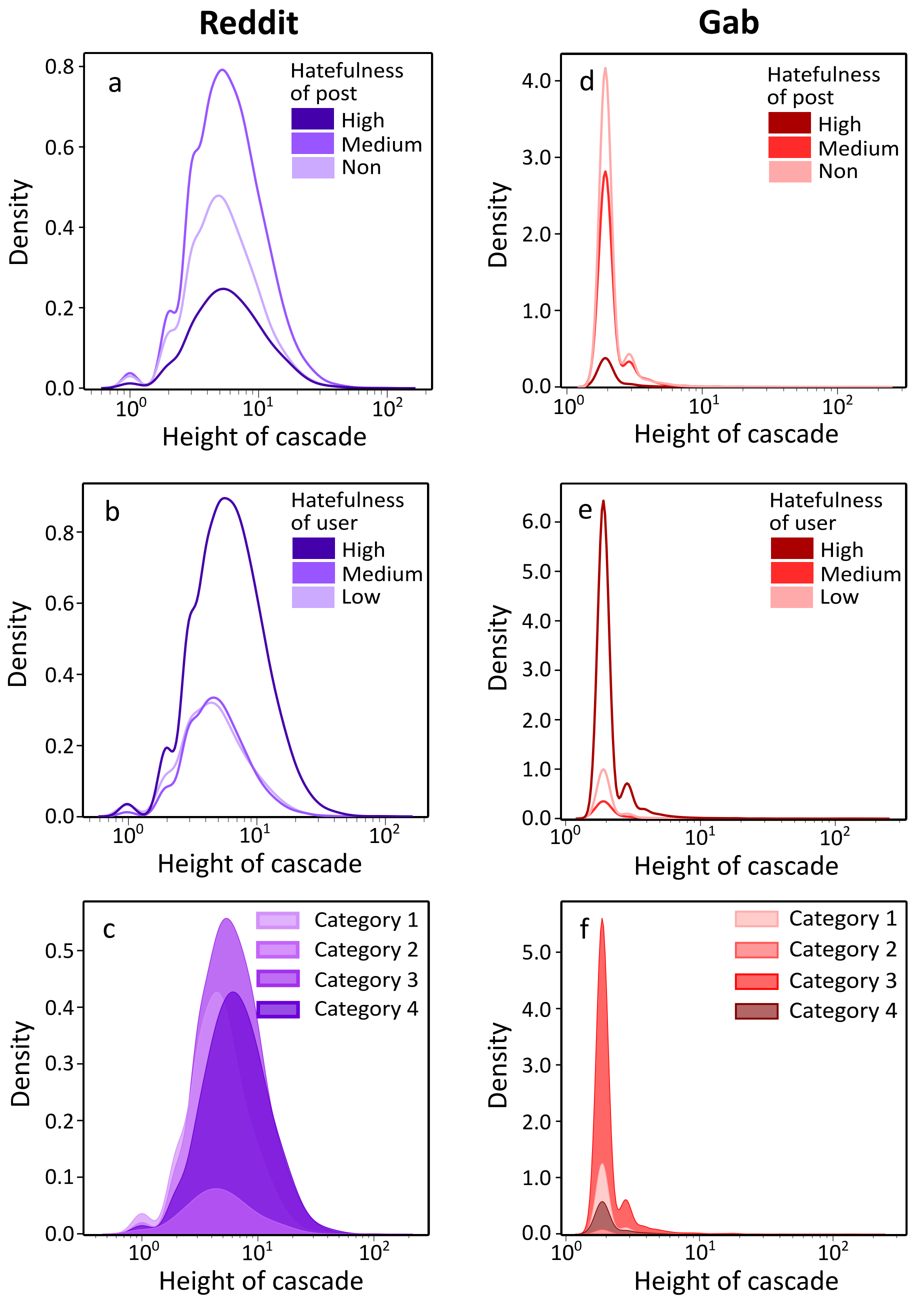

Fig. S5. Height distribution of cascades originating from hateful posts (subplots **a.**, **d.**) vs. posts from hateful users (subplots **b.**, **e.**); for a given value of cascade height in the x-axis, the corresponding y-value denotes the density of cascades corresponding to that height. For both Reddit and Gab, posts from highly-hateful users are more likely to produce cascades of larger height. We further present the height distribution of cascades originating from hateful users segregated on the basis of hateful posts (subplots **c.**, **f.**). Here, **Category 1** represents a low-hate post from a low-hate user, **Category 2** represents a high-hate post from a low hate user, **Category 3** represents a low-hate post from a high-hate user, and **Category 4** represents a high-hate post from a high-hate user. In all three networks, low-hate content posted by highly-hateful users tend to breed cascades with largest height. We do not present analysis of the height distribution of cascades for Twitter, since the dataset that we use does not show cascade height of more than 3.

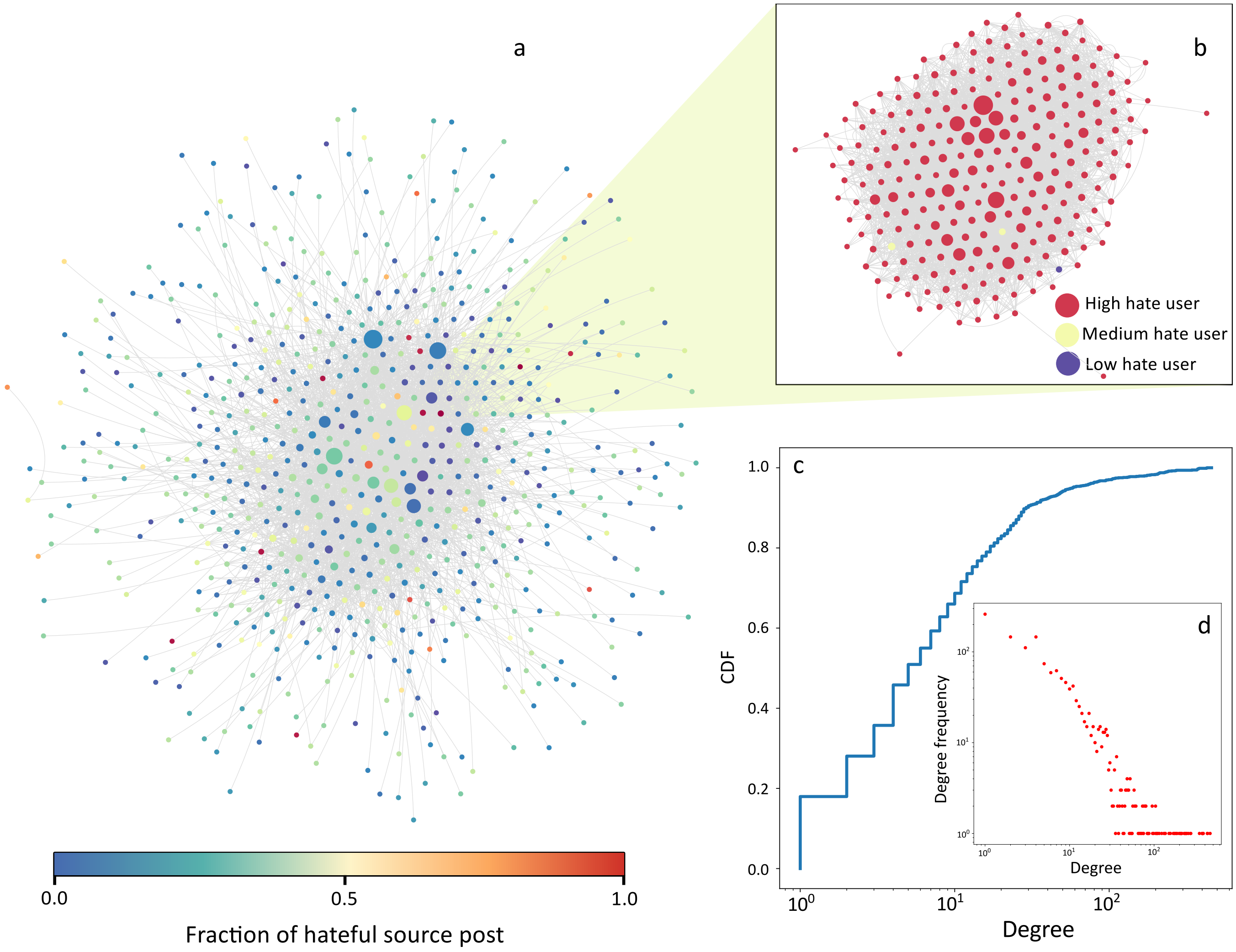

Fig. S6. Distribution of hate among echo chambers in Gab. **a.** A sample network of echo chambers; each node represents an echo chamber color coded with the fraction of hateful source posts posted by members of the echo chamber; an edge between two echo chambers denotes common users. **b.** A user interaction network within an example echo chamber; each node being a user with edges defined by *reply-to* interactions. **c.** and **d.** show the degree distributions of the network shown in **a.**

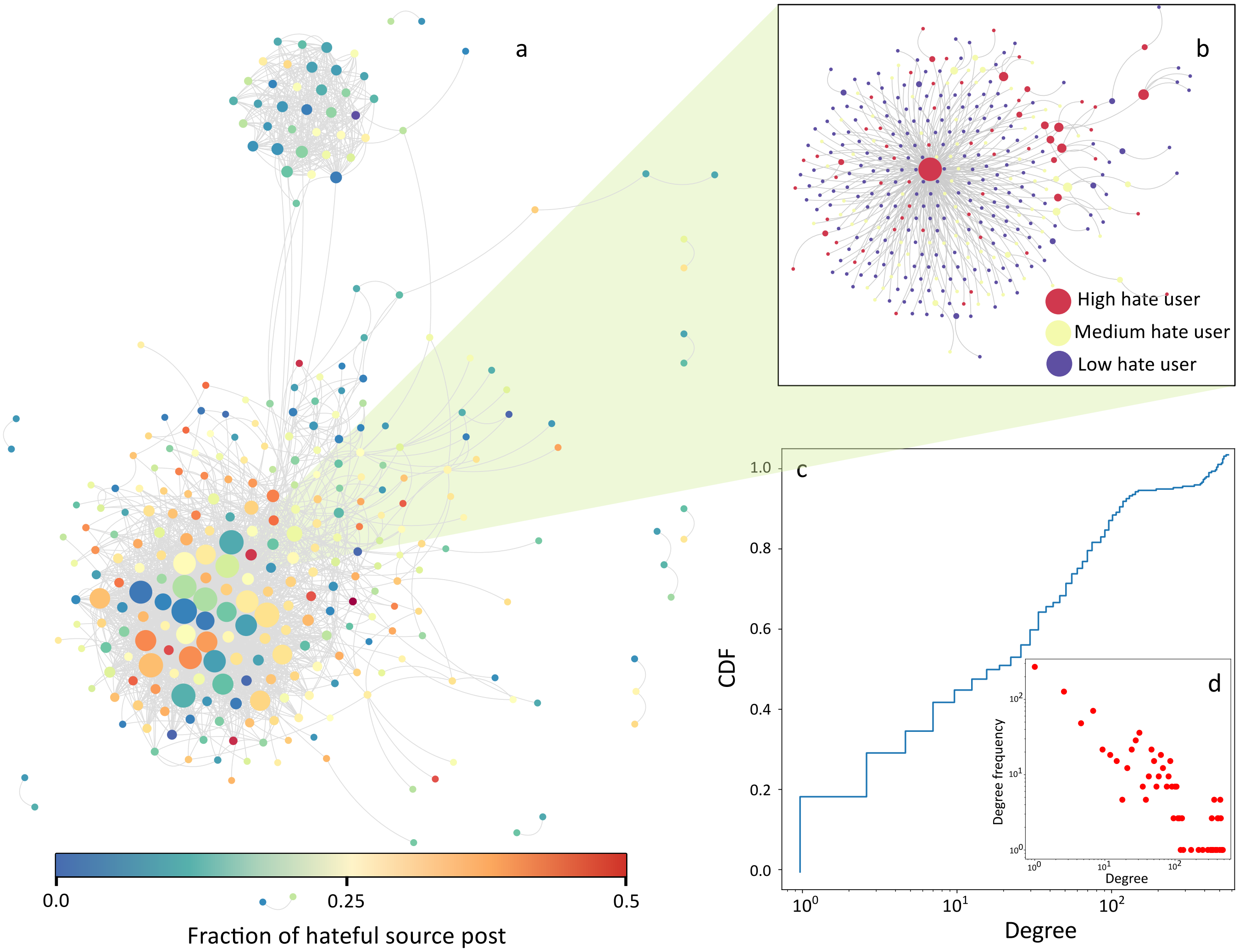

Fig. S7. Distribution of hate among echo chambers in Twitter. **a.** A sample network of echo chambers; each node represents an echo chamber color coded with the fraction of hateful source posts posted by members of the echo chamber; an edge between two echo chambers denotes common users. **b.** A user interaction network within an example echo chamber; each node being a user with edges defined by *reply-to* interactions. **c.** and **d.** show the degree distributions of the network shown in **a.**

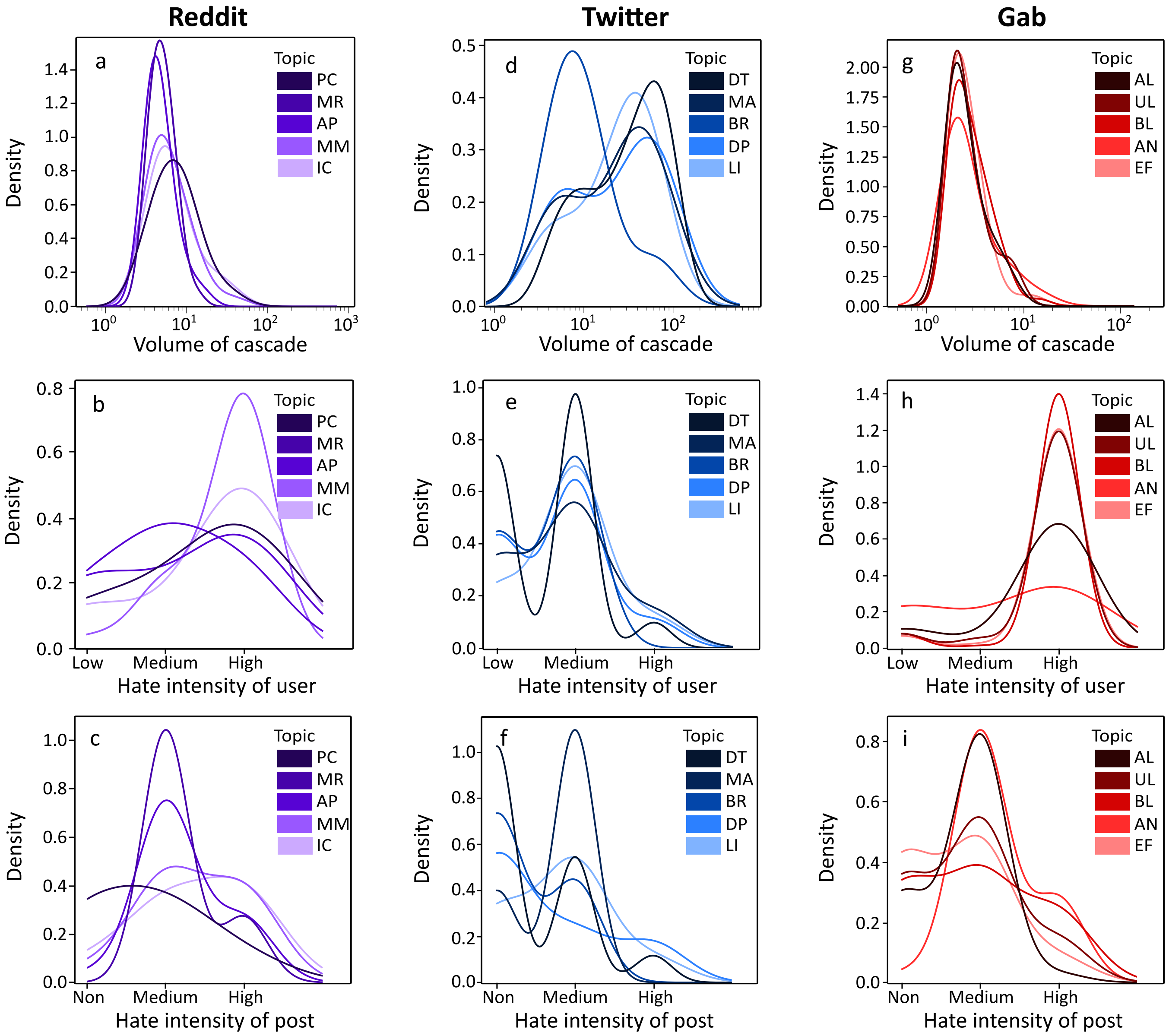

Fig. S8. Density distribution of hate intensity of source posts, source users as well as cascade volumes for top occuring topics in each platform. **a., d., g.** Cascade volume distribution for the three social network for the selected topics. **b., e., h.** Distribution of hate intensity of user for the three social netwokss across the selected topics. **c., f., i.** Distribution of hate intensity of source post for the three social networks across the selected topics. We cover five topics for each social network – Reddit (PC for pro-choice, MR for mens rights, AP for anti-abortion and pro-life, MM for mens mental health, IC for Illuminati conspiracy theories), Twitter (DT for Donald Trump, MA for MAGA, BR for Brexit, DP for Democratic Party, LI for #Liberal), and Gab (AL for anti-abortion laws, UL for US border laws, BL for black lives matter, AN for antisemitism, EF for election fraud).

References

1. M Cinelli, GDF Morales, A Galeazzi, W Quattrociocchi, M Starnini, The echo chamber effect on social media. *Proc. Natl. Acad. Sci.* **118**, e2023301118 (2021).
2. T Davidson, D Warmsley, M Macy, I Weber, Automated hate speech detection and the problem of offensive language in *ICWSM*. Vol. 11, (2017).
3. Z Waseem, D Hovy, Hateful symbols or hateful people? predictive features for hate speech detection on twitter in *Proceedings of the NAACL student research workshop*. pp. 88–93 (2016).
4. AM Founta, et al., A unified deep learning architecture for abuse detection in *WebSci*. p. 105–114 (2019).
5. B Mathew, R Dutt, P Goyal, A Mukherjee, Spread of hate speech in online social media in *Proceedings of the 10th ACM Conference on Web Science*, WebSci '19. (Association for Computing Machinery, New York, NY, USA), p. 173–182 (2019).
6. Z Zhang, L Luo, Hate speech detection: A solved problem? the challenging case of long tail on twitter. *Semantic Web* **10**, 925–945 (2019).
7. K Florio, V Basile, M Polignano, P Basile, V Patti, Time of your hate: The challenge of time in hate speech detection on social media. *Appl. Sci.* **10**, 4180 (2020).
8. D Cer, et al., Universal sentence encoder (2018).
9. D Cer, et al., Universal sentence encoder for english in *Proceedings of the 2018 conference on empirical methods in natural language processing: system demonstrations*. pp. 169–174 (2018).
10. L McInnes, J Healy, J Melville, Umap: Uniform manifold approximation and projection for dimension reduction (2018).
11. L McInnes, J Healy, S Astels, hdbscan: Hierarchical density based clustering. *J. Open Source Softw.* **2**, 205 (2017).
12. D Choi, S Chun, H Oh, J Han, TT Kwon, Rumor propagation is amplified by echo chambers in social media. *Sci. Reports* **10**, 310 (2020).
13. M Ángeles Serrano, M Boguñá, A Vespignani, Extracting the multiscale backbone of complex weighted networks. *Proc. Natl. Acad. Sci.* **106**, 6483–6488 (2009).
14. B Suh, L Hong, P Pirolli, EH Chi, Want to be retweeted? large scale analytics on factors impacting retweet in twitter network in *2010 IEEE Second International Conference on Social Computing*. pp. 177–184 (2010).
15. E Bakshy, JM Hofman, WA Mason, DJ Watts, Everyone's an influencer: Quantifying influence on twitter in *Proceedings of the Fourth ACM International Conference on Web Search and Data Mining*, WSDM '11. (Association for Computing Machinery, New York, NY, USA), p. 65–74 (2011).
